# Stability in plant–pollinator communities across organizational levels: present, gaps, and future

**DOI:** 10.1093/aobpla/plae026

**Published:** 2024-05-20

**Authors:** Ainhoa Magrach, Daniel Montoya

**Affiliations:** Basque Centre for Climate Change (BC3), 48940 Leioa, Spain; Ikerbasque, Basque Foundation for Science, 48011 Bilbao, Spain; Basque Centre for Climate Change (BC3), 48940 Leioa, Spain; Ikerbasque, Basque Foundation for Science, 48011 Bilbao, Spain

**Keywords:** Ecosystem function, organizational level, plant-pollinator interactions, stability, trophic level

## Abstract

**Abstract**. The study of ecological stability continues to fill the pages of scientific journals almost seven decades after the first ecologists initiated this line of research. The many advances in this field have focused on understanding the stability of populations, communities or functions within single guilds or trophic levels, with less research conducted across multiple trophic levels and considering the different interactions that relate species to each other. Here, we review the recent literature on the multiple dimensions of ecological stability specifically within plant–pollinator communities. We then focus on one of stability´s dimensions, temporal invariability, and adapt an existing partitioning framework that bridges invariability and synchrony measures across spatial scales and organizational levels to accommodate interactions between plants and their pollinators. Finally, we use this framework to analyse temporal invariability in plant reproductive success, partitioning it on invariability and synchrony components across plant and pollinator populations and communities, as well as their interactions, using a well-resolved dataset that encompasses data for two years. Our review of the literature points to several significant gaps in our current knowledge, with simulation studies clearly overrepresented in the literature as opposed to experimental or empirical approaches. Our quantitative approach to partitioning invariability shows similar patterns of decreasing temporal invariability across increasing organizational levels driven by asynchronous dynamics amongst populations and communities, which overall stabilize ecosystem functioning (plant reproductive success). This study represents a first step towards a better comprehension of temporal invariability in ecosystem functions defined by interactions between species and provides a blueprint for the type of spatially replicated multi-year data that needs to be collected in the future to further our understanding of ecological stability within multi-trophic communities.

## Teaser

As global environmental changes increasingly impact ecosystems worldwide, preserving ecosystem functions and stability becomes crucial for biodiversity conservation. Despite decades of research, much focus has been on the stability of species, communities, and functions within specific ecological niches, with less attention to interactions across multiple trophic levels. This review highlights recent literature on stability within multi-trophic plant and pollinator communities, revealing biases toward simulation studies over empirical validation. It adapts a framework to analyse stability and synchrony across organizational scales, emphasizing the importance of interaction frequencies in understanding temporal stability in plant reproductive success. The study underscores the necessity for spatially replicated, multi-year datasets encompassing various community components to advance our comprehension of ecosystem stability. This work marks an initial stride towards understanding the temporal stability of species interactions, offering insights into future research directions for ecosystem conservation and management.

## Introduction

Ecological stability has been a central topic in Ecology for the past decades, a long tradition that dates back to the early 50s of the 20th century when different researchers attempted to reveal the drivers of community stability, and specifically the relationship between diversity and stability (MacArthur 1955; May 2001). The initial, intuitive hypothesis was that complexity begets stability—i.e. that more complex communities should be more stable (MacArthur 1955; Elton 1958; Odum 2017). This hypothesis was later challenged by May (1972), who used randomly simulated networks and local stability analysis to conclude that, on the contrary, more complex communities were indeed unstable. These contrasting paradigms triggered multiple theoretical and empirical studies that have stimulated the so-called complexity (diversity)—stability debate (McCann 2000).

While our understanding of community stability has advanced significantly in the last decades (Tilman 1995; Brose *et al.* 2006; Gravel *et al.* 2011), a general comprehension of ecological stability has been hindered by a wealth of different definitions and metrics used to measure it across multiple spatial and temporal scales, and organizational levels (Kéfi *et al.* 2019). Ecological stability (see Box 1) is a *multidimensional* concept, that encompasses at least four different properties: temporal invariability, persistence, resistance and resilience (Bello *et al.* 2021). The outcome of these different dimensions, or components, of stability depends on several factors, such as (i) the element for which stability is being measured in the system (e.g. biomass or species composition), (ii) the organizational level considered (e.g. populations *versus* communities), or (iii) the existence and type of perturbations (e.g. droughts can reduce plant biomass while maintaining a similar plant species composition, yet the impacts of invasive species may target species composition more strongly than plant biomass), amongst others. Therefore, any meaningful statement about the stability of a system should clearly identify the element(s) being measured, the type of perturbation considered (if any), the stability dimension being observed (e.g. temporal invariability), and the organizational level that is being considered (Donohue *et al.* 2013; Arnoldi *et al.* 2016; Radchuk *et al.* 2019).

Box 1. **Definitions of concepts**

- Ecological stability: a multi-faceted concept that includes the ability of an ecosystem to minimize the variability over time of one of its elements (e.g. temporal invariability) or to recover after a perturbation (e.g. persistence, resistance, resilience, reviewed in Bello *et al.* 2021).
- Persistence: The length of time a system maintains a certain reference condition (Van Meerbeek *et al.* 2021).
- Resilience: The rate at which a system variable returns to its reference condition following a perturbation (Van Meerbeek *et al.* 2021).
- Resistance: The ability to resist changes in system variables in response to a perturbation. Resistance is inversely related to the degree of change following a perturbation (Van Meerbeek *et al.* 2021).
- Invariability: The magnitude of fluctuations of a system variable around its mean value. Calculated as the inverse of the Coefficient of Variation (1/CV), which is the ratio of the standard deviation to the mean. The higher the coefficient of variation, the greater the level of dispersion around the mean, and the larger the variability (i.e. lower stability). Invariability is suggested to be an integrative metric because while resistance describes the immediate effect of disturbances on a system variable (e.g. abundance), and resilience describes the rate at which abundances recover from disturbances, invariability describes the joint effects of these two processes on dynamics over time (Clark *et al.* 2021).
- Spatial synchrony: correlation in fluctuations of multiple species’ abundances across different spatial locations or communities (Wang *et al.* 2019a).
- Species synchrony: correlation in fluctuations of multiple species’ abundances within a community (Wang *et al.* 2019a).

Despite such complexity, several efforts have been made to synthesize the multidimensionality of stability, either theoretically (Domínguez-García *et al.* 2019) or empirically (Donohue *et al.* 2013). This research shows that many of the stability metrics used in the literature are correlated (Domínguez-García *et al.* 2019), collapsing into a smaller number of independent components. However, these studies have also detected a series of limitations in the analysis of the stability of ecological systems. For example, in their review of the literature Kéfi *et al.* (2019) found a significant bias towards measuring stability at the community level, with less effort placed at smaller (e.g. local population) or larger scales (e.g. regional), and even less research conducted across organizational levels (Wang *et al.* 2019a).

**Figure 1.**
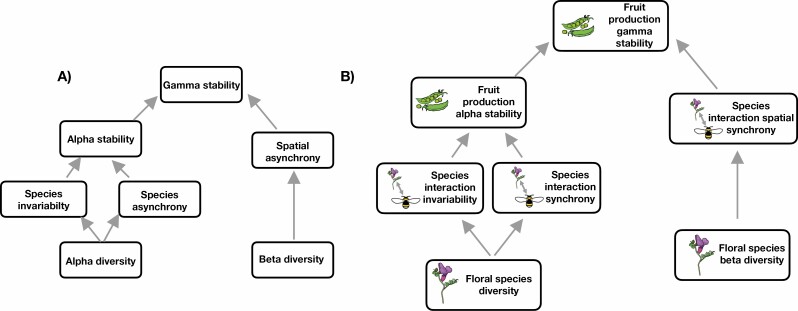
Conceptual diagram showing the potential paths and mechanisms by which diversity, stability and synchrony across different trophic levels could affect final functions across different organizational levels. (A) Framework developed for single trophic levels, showing how diversity affects local species stability and asynchronies, how these in turn affect local community stability and how the combination of species turnover and spatial asynchronies with local community stability affect regional stability (diagram modified from Fig. 1e in Hautier *et al.* 2020). (B) Modification of the ‘classical’ framework to accommodate multiple trophic levels, specifically plant–pollinator interactions and their effect on plant reproductive success across organizational levels. Here, floral species diversity is expected to have direct impacts on plant–pollinator interaction stability and synchrony. Together, these two components are expected to affect local fruit production. Turnover in floral species composition (beta-diversity) is expected to affect interaction spatial synchrony, which in conjunction with local fruit production will affect regional fruit production. Here, we include solely plant–pollinator interactions and not pollinator abundances because interactions reflect information on species abundances but also other factors that affect their realization (e.g. phenology).

Although empirical evidence suggests that stability is not necessarily correlated positively at different organizational levels, and that seemingly unstable populations result in stable communities (Tilman 1995; Xu *et al.* 2021), a lack of a theoretical framework to quantify the processes that determine the stability of ecosystem functions beyond the community level (i.e. regional metacommunity) has hampered our advances in the cross-scale understanding of stability (with a few exceptions, e.g. Anderson *et al.* 2013; Segrestin *et al.* 2024). This becomes particularly complex as connectivity within metacommunities can result in non-linearities that affect stability and synchrony values across populations and communities (Gonzalez *et al.* 2020). These issues have been, at least partially, solved with the development of a new theoretical framework that has identified the different mechanisms that define stability at any given scale (Wang and Loreau 2014, 2016; Wang *et al.* 2019a), thus allowing us to quantify these processes and scale them from populations, to communities, to regional scales/metacommunities. Specifically, within this framework the stability of a local community (*alpha stability*) is driven by two processes: *species stability* and *species synchronies*, each of which can be determined by local species diversity (*alpha diversity*, Fig. 1A). In turn, regional or higher-level stability (*gamma stability*) is determined by alpha stability and *spatial synchronies*, i.e. the asynchronous dynamics amongst local communities (Box 1), which are also driven by species turnover, i.e. *beta-diversity* (Fig. 1A). As such, there are different combinations that can lead to an overall stability at the regional level (Box 2).

Box 2. **A mechanistic theory of the drivers of stability (invariability) across spatial scales**

A recent mechanistic framework has been developed to quantify ecological stability across spatial scales and organizational levels (Wang and Loreau 2014, 2016; Wang *et al*. 2019a), largely rooted on the biodiversity insurance hypothesis (Yachi and Loreau 1999). Stability is defined as the temporal invariability of a system variable (e.g. productivity) at any given scale. Higher local scale community stability (i.e. alpha stability) can be determined by two processes, namely species stability and species asynchrony. On one hand, a higher mean temporal stability of all species within the community (i.e. species stability) can have a stabilizing effect due to a lower inter-annual variation in single species’ abundances. On the other hand, more asynchronous temporal dynamics among species in response to environmental fluctuations (i.e. species asynchrony) can also be stabilizing if compensatory dynamics are in place; that is, if reductions in the abundance of some species through time are compensated by increases in other species. At larger scales (i.e. gamma stability), higher stability can be driven by higher alpha stability and more asynchronous dynamics across local communities (i.e. spatial asynchrony). Therefore, the stabilizing effect of species asynchrony at the local scales (i.e. local/species insurance hypothesis) parallels the stabilizing effect of spatial asynchrony at larger spatial scales (i.e. spatial insurance hypothesis). Higher species diversity at the local scale (i.e. alpha diversity) can result in larger species synchrony and species stability, whereas higher local species diversity and/or greater variation in species composition across communities (i.e. beta-diversity) can result in larger spatial asynchrony.

Beyond the efforts aimed at understanding stability across organizational levels, there are still other aspects that remain unexplored. Specifically, most research has focused on single trophic levels within experimental settings, particularly primary producers like plants (Tilman 1995), and a large knowledge gap appears in our understanding of stability within communities involving more than one level, such as those involving plants and their pollinators (Loreau *et al.* 2021). A major challenge when moving to multiple trophic levels includes accounting for the complex networks of interactions that link these levels. This step, a small step conceptually, represents a giant leap methodologically. Further, beyond considering the interactions between trophic levels and how these affect the stability of each individual level, the outcomes of these interactions (e.g. reproductive success of both the plant side and the pollinator side of the interaction) also need to be accounted for and their stability assessed. This is particularly needed in the case of the pollinator side of the interaction, for which performance as a consequence of their interactions with plants is largely ignored.

Although the bulk of studies on stability within complex communities has focused on single trophic levels, some research has started to address stability in communities with more than one trophic level (e.g. Lázaro *et al.* 2022; Tobajas *et al.* 2023). Overall, these studies reveal that ecological stability within these multi-trophic systems depends on factors such as the trophic level/group considered (Firkowski *et al.* 2021; Siqueira *et al.* 2023), the spatial scale of analysis (Siqueira *et al.* 2023), landscape heterogeneity (Lázaro *et al.* 2022), habitat fragmentation (Ren *et al.* 2022), the diversity and proportion of interaction types (Lurgi *et al.* 2015) and the structure of species interactions networks (Neutel *et al.* 2002; McWilliams *et al.* 2019; Duchenne *et al.* 2022; Lázaro *et al.* 2022; Nie *et al.* 2023). However, while some of these studies focus on mutualistic communities (e.g. Duchenne *et al.* 2022; Lázaro *et al.* 2022), few have simultaneously combined the study of multi-trophic interactions with measures of the stability of interaction-dependent ecosystem functions (e.g. fruit set in plant–pollinator communities) in empirical plant–pollinator communities across different spatial scales.

The work we present here has two main goals. Our first goal is to develop a systematic map (James *et al.* 2016) of the recent literature on ecological stability within plant–pollinator communities, that is used to assess all the different stability dimensions covered by these studies, as well as their main results. Following this systematic map, we identify research gaps that require further scientific attention. Our second goal is to provide a proof of concept where we use a plant–pollinator dataset that includes data on floral resource production, pollinator visitation rates, plant–pollinator interaction frequencies and plant reproductive success values collected across different organizational levels (from populations to communities to regions) to illustrate how stability scales across spatial scales and organizational levels. Specifically, we focus on temporal invariability as a measure of stability for various reasons: (i) the synchrony-stability framework is based on invariability, (ii) it is an integrative measure of stability (Loreau *et al.* 2021), frequently used in ecology (Tilman *et al.* 2006; Donohue *et al.* 2016), as it describes the combined effects of resistance and resilience on temporal community dynamics after perturbations (Clark *et al.* 2021) and can be easily quantified in both theoretical and empirical approaches, and (iii) it is the most commonly used stability metric in empirical studies such as this one (Donohue *et al.* 2016; Craven *et al.* 2018; Wang *et al.* 2019b; Hautier *et al.* 2020). To this end, we use the partitioning framework developed Wang *et al.* (2019a), which integrates stability and synchrony measures across spatial scales and organizational levels. We calculate temporal invariability at the population, community and regional levels for flower production, pollinator visitation rates, and interaction frequencies, and assess how temporal invariability scales across organizational levels for each of these system variables.

## Stability in Plant–Pollinator Community Studies: A Systematic Map

### Methodology

We surveyed the recent literature to assess the extent to which stability and its multiple dimensions within plant–pollinator communities have been studied in the past decades. To this end, we extracted literature from Web of Science based on the query string TS = ((stability OR resilience OR persistence OR resistance) AND pollinat* AND (community OR diversity OR evenness OR richness)). From here, we obtained 1552 references. We then assessed paper abstracts to reduce our selection to papers that had specifically measured one of the dimensions of stability, ignoring other types of papers, such as reviews or perspectives on the topic. When crop productivity was reported we only focused on studies analysing pollinator-dependent crops. Our final selection included 71 papers (see flow chart in Supplementary Appendix 2 Fig. S1, Haddaway *et al.* (2022), and Supplementary Appendix 1). For each of these, we extracted a number of variables including, (i) the dimension of stability considered: temporal invariability, persistence, resistance or resilience (ii) the number of stability metrics recorded, (iii) the type of metric used to define stability (e.g. the coefficient of variation, CV), (iv) the organizational level at which this variable was recorded (population, community, region), (v) the spatial scale, (vi) the number of replicates, (vii) the temporal scale, (viii) the temporal resolution, (ix) and the type of study (simulation, empirical, experimental, see Supplementary Appendix 1 for full dataset).

### Main results and discussion

**Figure 2.**
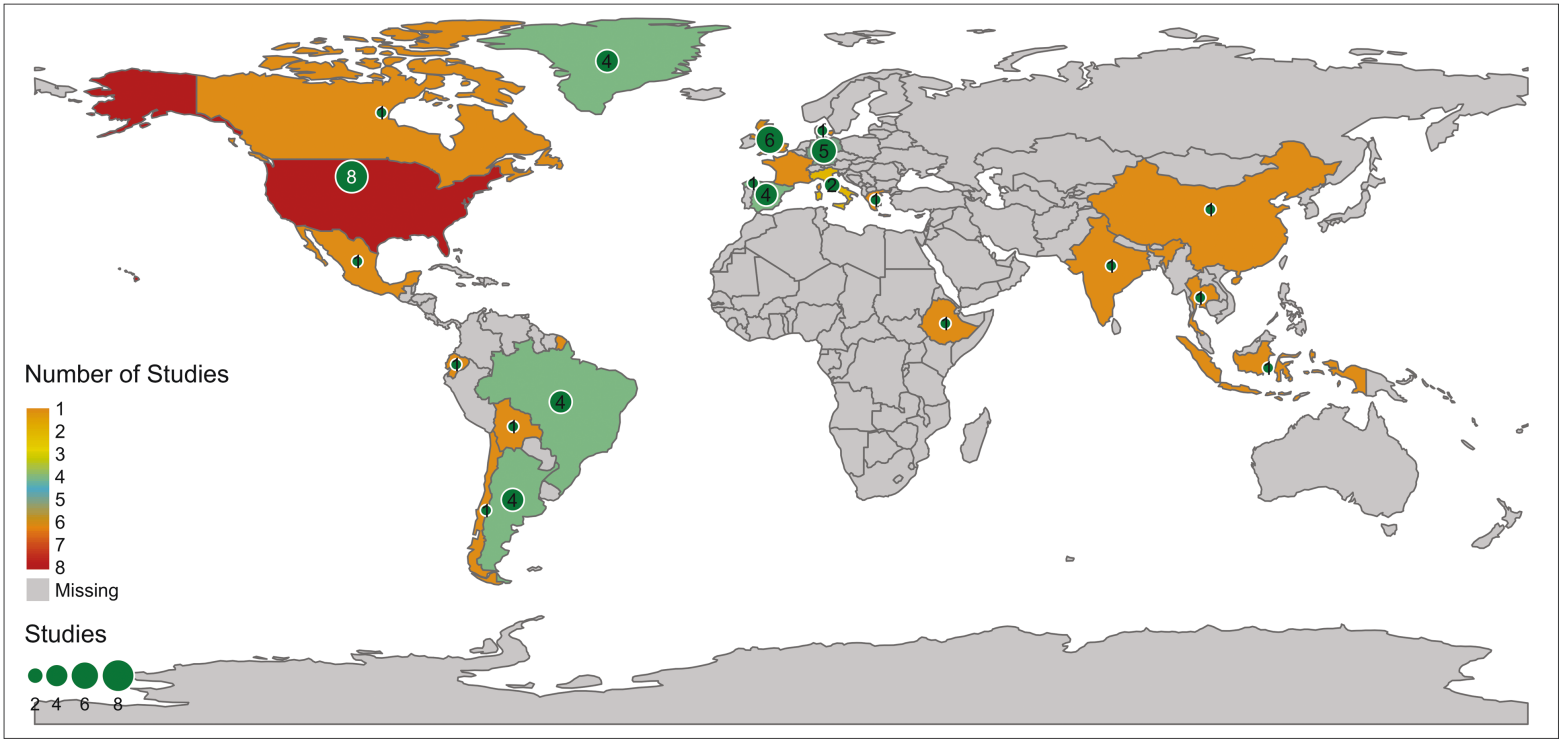
Geographical distribution of the number of studies analysing at least one component of stability within plant–pollinator communities.

**Figure 3.**
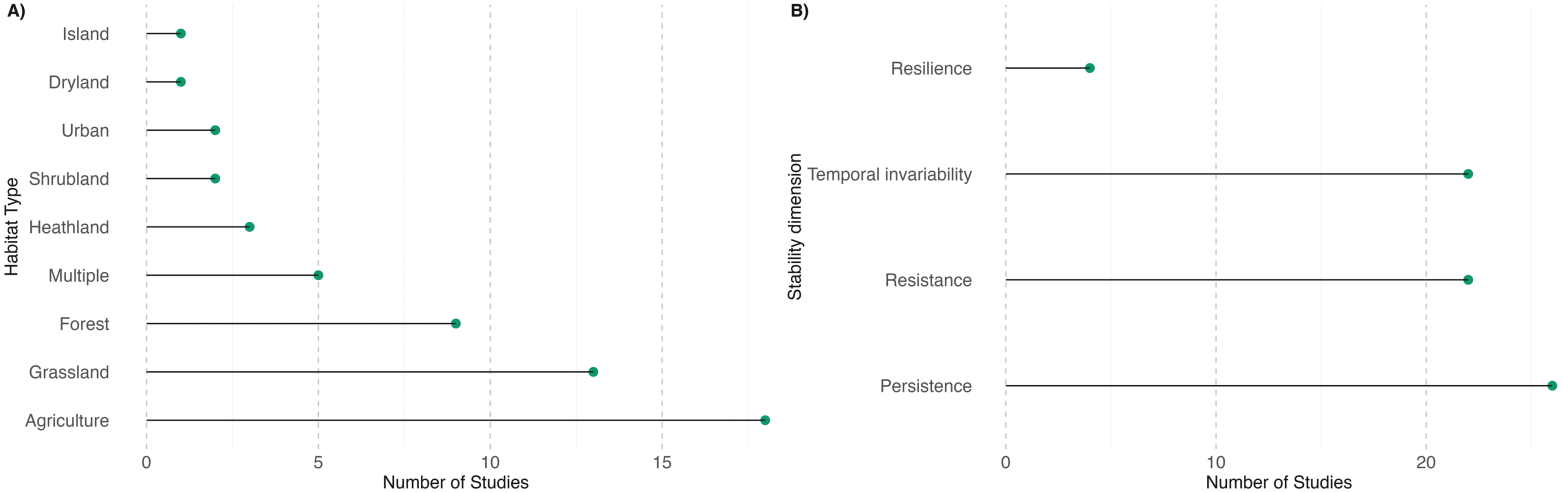
Rank abundance plots showing (A) the distribution of studies amongst different habitat types and (B) the stability dimension considered within each study.

Our systematic map shows that studies have been conducted within 22 countries (Fig. 2), or combine information for larger regions (e.g. European Union or global scales, *N* = 1 and *N* = 6, respectively). There is a significant bias in the location of studies: particularly abundant within the USA, several European countries (e.g. UK, Germany and Spain) and regions (e.g. Greenland), and two South American countries: Argentina and Brazil. There is a small representation of single studies across some Asian countries and practically no data from African countries. A significant portion of the studies are based on simulations and not tied to specific regions (*N* = 16). In terms of habitats, most studies correspond to agricultural landscapes, followed by grasslands and different types of forests (Fig. 3A).

In the case of plant–pollinator communities, most research has focused on three dimensions of stability and on a few metrics to measure each of them. This contrasts with previous, more general reviews of the literature (Kéfi *et al.* 2019), which found a multitude of different stability metrics and dimensions. Specifically, the most studied dimension is persistence (25 studies), followed by temporal invariability (22 studies), and resistance (22 studies, Fig. 3B). The metrics most commonly used to measure these dimensions include the proportion of remaining species or interactions in the case of persistence, and the coefficient of variation (CV) of a specific system variable in the case of temporal invariability. In the case of resistance, a particularly popular metric is robustness, which measures the probability of secondary extinctions within the plant or pollinator level following the loss of their partner species (Memmott *et al.* 2004).

**Figure 4.**
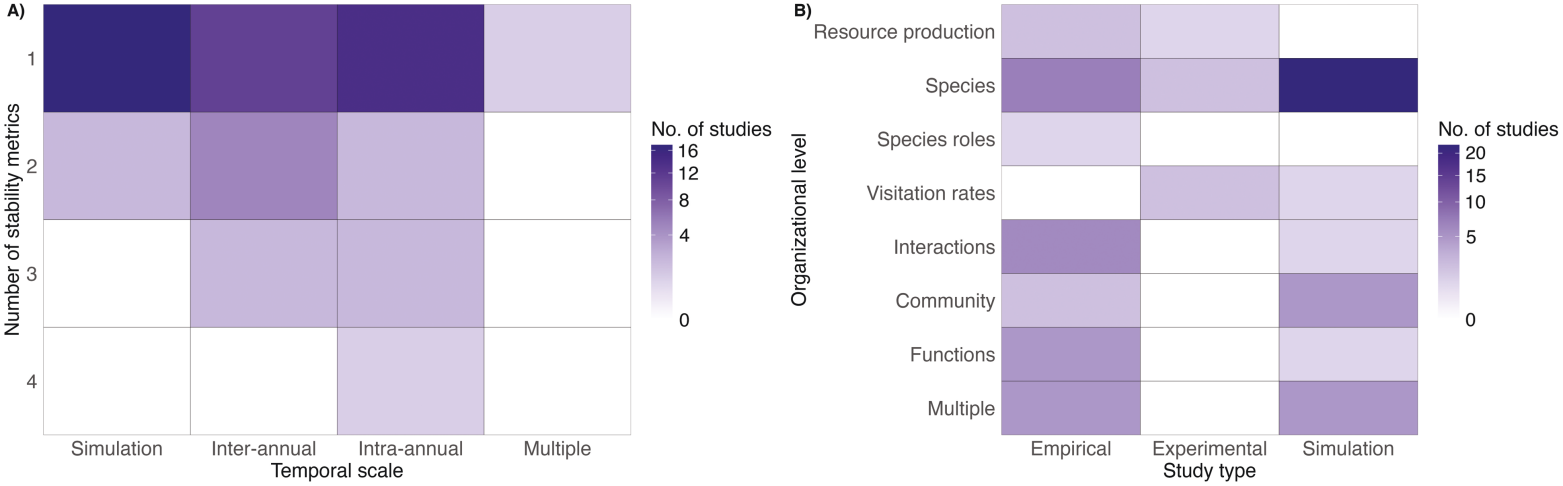
Results of the systematic map on the stability of plant–pollinator communities. Heatmaps show (A) the number of stability metrics, temporal scale and (B) organizational level and type of study most often used in the recent literature focusing on the stability of plant–pollinator communities. Darker colors indicate larger number of studies focusing on a specific aspect and white areas indicate significant gaps in the literature.

Most of the studies included in our review have been conducted either at relatively small spatial scales, such as the site level (39 studies), and far fewer studies at landscape (10 studies) or larger scales (e.g. national levels, with three studies focusing on crop yield stability or pollinator community stability across France and the UK). The great majority of studies have focused on a single measure of stability (50 studies) and are either the result of simulations with no specified temporal or spatial scale or based on intra-annual measures of stability (Fig. 4A). In terms of organizational level, the largest number of studies has focused on measures of stability at the level of populations within single species (31 studies), with fewer research focusing on multiple organizational levels (13 studies), or other levels, such as interactions (7 studies), or the functional outcomes of these interactions (6 studies, Fig. 4B).

Most research on stability within plant–pollinator communities is based on completely simulated communities or on simulated perturbations to real communities (35 studies), followed by empirical research (28 studies) and very few experimental contributions (5 studies, Fig. 4B). Amongst all the studies considered, 62 % focused on a measure of stability related to a specific perturbation, as opposed to 38 % of the studies that focused on understanding the underlying stability of a system in response to environmental fluctuations (e.g. focusing on temporal invariability).

Our literature review indicates that plant–pollinator interactions vary yearly, with a core set persisting while many are infrequent (Chacoff *et al.* 2017). These dynamics relate to changes in species abundances and roles (Cirtwill *et al.* 2018; Silva Goldas *et al.* 2021). Species with overlapping phenologies and matching traits tend to have more stable relationships (Peralta *et al.* 2020). While some studies suggest generalists persist better due to environmental tolerance (Resasco *et al.* 2021), others argue their abundance, not diet breadth, drives persistence (Winfree *et al.* 2014).

Regarding perturbations, our review shows that introduced species like honeybees can enhance network stability (Corcos *et al.* 2020), but may alter the diet of local pollinators (Magrach *et al.* 2017). Keystone species can sometimes bolster resistance to invasives (Vitali *et al.* 2021), while urbanization often correlates with greater community resistance, likely due to increased redundancy in species roles (Cortina *et al.* 2022). However, this redundancy may fluctuate during flowering seasons (Fantinato *et al.* 2018; Guzman *et al.* 2021), rendering communities more vulnerable. While more nested communities generally show increased stability (Memtsas *et al.* 2022), they can still be adversely affected by perturbations like drought, which further undermine their robustness (Lance *et al.* 2017; Rabeling *et al.* 2019).

In general, studies find a positive biodiversity-stability relationship, particularly in the case of floral production (Dorado and Vázquez 2014; Cong *et al.* 2020). These positive relationships are also found between plant species diversity and the stability of pollinator communities (Senapathi *et al.* 2021), and pollinator visitation rates (Ebeling *et al.* 2008, 2011). Within agricultural landscapes, management practices that enhance the presence of floral resources through time, such as maintaining hedgerows (M’Gonigle *et al.* 2015; Gardner *et al.* 2021), nectar-rich gardens (Tew *et al.* 2022), greater landscape heterogeneity (Papanikolaou *et al.* 2016; Lázaro and Alomar 2019; Martínez-Núñez *et al.* 2019), increasing the connectivity of natural habitats (Montoya *et al.* 2019), or reducing the distance to natural areas (Klein 2009; Garibaldi *et al.* 2011; Sritongchuay *et al.* 2019; Montoya *et al.* 2021) also ensure stable pollinator populations (Gardner *et al.* 2021) and greater stability in productivity (Geeraert *et al.* 2020). In some cases, this positive relationship is not found as it was mostly common abundant and generalist species driving ecosystem services, suggesting that selection effects, where particular species with key traits disproportionately influence ecosystem functioning, may not always align with patterns of species abundance (Genung *et al.* 2017; Redhead *et al.* 2018). In turn, restoration efforts reveal complexities as multiple trophic levels are considered: plants benefit from restoration and invasive plant removal, while pollinators suffer from floral resource loss (Valdovinos *et al.* 2009; Gaiarsa and Bascompte 2022).

Four key takeaways emerge from this review. First, numerous research gaps remain, especially concerning stability’s various dimensions, its scaling across organizational levels, and its spatial dynamics (observe the blank spaces within our heat maps in Fig. 4A and B). Crucially, experimental tests of existing theory, validated with empirical data, are lacking. Secondly, much knowledge stems from computer simulations of either entirely artificial communities or perturbations within real ones, lacking empirical validation (e.g., research on robustness to species extinctions). Robustness has been central in plant–pollinator network studies since Memmott *et al*’.s seminal work (2004), with advancements including probabilities of rewiring post-species loss (Kaiser-Bunbury *et al*. 2010; Baldock *et al*. 2019) and different loss patterns and rewiring potentials (Ávila-Thieme *et al*. 2023). Yet, our understanding of how perturbations affect trophic levels will stagnate without validating simulation results with empirical data. Thirdly, the majority of research has focused on single organizational levels, notably populations, leaving gaps in understanding stability’s scaling across higher levels (e.g. communities, though exceptions exist, such as Lázaro *et al.* 2022; Tobajas *et al.* 2023). In contrast, other systems like plants and biomass production have extensively explored how stability scales with organizational levels driven by various factors (Tilman 1995; Wang *et al.* 2019a). Finally, fourthly, this review underscores that mechanisms affecting local and regional stability within single trophic levels, such as species population synchronies (Wang *et al.* 2019a), are rarely considered in the context of species interactions (though exceptions exist, like Lázaro *et al.* 2022). While synchronous dynamics are well-studied for single species or communities, their implications for species interactions remain largely unexplored, despite potential impacts on ecosystem function stability. Future research should adopt a holistic approach to understanding stability in plant–pollinator communities, integrating information from floral resource production to community diversity and abundance, and ultimately ecosystem functions. Methodological challenges can be addressed by adapting existing frameworks for single trophic levels (e.g. Wang *et al.* 2019a; Hautier *et al.* 2020).

## A Framework to Study Stability Within Plant–Pollinator Communities: The Case of Temporal Invariability

Recent advances have developed a framework to partition the stability of ecological functions, specifically their temporal invariability, at the level of meta-communities by bridging variability and synchrony measures across spatial scales and organizational levels (Wang and Loreau 2014; Wang *et al.* 2019a; Box 2). Here, temporal invariability at the regional (metacommunity) scale is decomposed into the product of population variability, and population species and spatial synchrony metrics, to simultaneously account for spatial scale and organizational level. Traditionally, this framework has been applied to study single trophic levels, particularly primary producers like plants and the stability of their biomass as an ecosystem function (e.g. Hautier *et al.* 2020, Fig. 1A). The next step is to adapt this framework to consider multiple trophic levels, and to partition the temporal invariability of functions that arise as a consequence of the interactions between these trophic levels (e.g. plant reproductive success arising from interactions between plants and their pollinators). In this direction, Liang *et al.* (2021) studied the effect of herbivory on the temporal invariability in grassland biomass productivity at multiple spatial scales; yet, this work did not consider species interactions between trophic levels explicitly (e.g. ignoring herbivore diversity or the frequency of herbivore-plant interactions).

Here, we contend that variability in species interactions is fundamental to explaining ecosystem function stability. This is based on the fact that many ecosystem functions depend on interactions between species from two trophic levels (e.g. pollination, seed dispersal, biotic control) and thus, higher stability of interactions is expected to yield more stable ecosystem functioning. Further, although species interactions are associated with population abundances, realized or observed interactions between species also depend on other factors, such as phenology, spatiotemporal distribution and co-occurrence of interaction partners or trait matching, among others (Vázquez *et al.* 2009; CaraDonna *et al.* 2017). Therefore, species interactions provide a more informative link to many ecosystem functions and their stability.

In the case of plant–pollinator communities, adapting this framework requires relating diversity values across the two trophic levels and their interactions, to population variability and synchrony measures, scaling across organizational levels, spatial scales and, additionally, trophic levels. This scaling can be done by connecting measures of floral species diversity, which are related to more stable values of floral resource production (Dorado and Vázquez 2014), to the stability and synchrony of both pollinator populations and their interactions with plants (Fig. 1B). Together, stability and synchrony values of both individual pollinator species and their interactions with plants can determine local population and community stability in fruit set (Fig. 1B). Spatial turnover (beta-diversity) in floral resources can lead to spatial asynchronies in both pollinator species and their interactions with plants (Magrach *et al.* 2023), and ultimately affect the stability of plant reproductive success (a measure of ecosystem function resulting from plant–pollinator interactions) across different organizational levels, from local species and community values of stability, to regional stability levels (Fig. 1B).

Part of this ladder towards an understanding of stability within multi-trophic communities has recently started to be climbed in the case of plant–pollinator communities (e.g. Lázaro *et al.* 2022; Tobajas *et al.* 2023). This research includes intra and inter-annual measures of stability of plant–pollinator species population abundances, their interactions and the structure of these communities. Despite this progress, several aspects must be added to fill the whole framework. Specifically, next steps should (i) consider how stability scales across organizational levels (from populations to communities to regional levels), (ii) evaluate how species and community stability values simultaneously and ultimately affect ecosystem function, and/or (iii) assess how turnover in both species and interactions through space (e.g. Magrach *et al.* 2023) interact to shape functional stability across larger spatial scales. This requires an intense level of sampling, requiring multiple study years, locations and well-resolved data on plant–pollinator interactions and resulting plant reproductive success. Engaging in such efforts is essential for us to initiate an exploration into various dimensions of stability within multi-trophic communities.

## Inter-Annual Temporal Invariability Across Organizational Levels, Spatial Scales and Trophic Levels: A Proof of Concept

To pave the way into filling some of the above-mentioned gaps, we use a dataset that includes biweekly samplings across the whole flowering season (February–May, *N* = 8 sampling periods per year) for two years, which we combine to assess inter-annual temporal invariability in fruit set, and how it is determined by different variability and synchrony measures across organizational levels, spatial scales and trophic levels. The dataset includes measures of floral resource production, pollinator visitation rates, interaction frequencies and plant reproductive success. Our study area is located within the vicinity of Doñana national park in SW Spain, an area with a Mediterranean climate characterized by warm dry summers and cool humid winters. Annual precipitation is 500 mm, and during the floral period (February–May), mean temperatures range from 12.5 to 22.5°C (Pizarro *et al.* 2021). We conducted all our surveys within five stone pine (*Pinus pinea*) woodland fragments, that include a rich understory of flowering shrubs and annual plants (Aparicio 2007), each one separated from the rest by at least 3 km (Supplementary Appendix 2 Fig. S2) during 2020 and 2021. Climatic data for both years were very similar (minimum temperature: 17.21 and 16.9°C, mean temperature: 11.58 and 11.17°C, maximum temperature: 22.82 and 22.62°C, precipitation: 182.4 and 110.4 mm, respectively for 2020 and 2021), data extracted using package *climaemet* (Pizarro *et al.* 2021). Within each site, we established a 20 × 20 m square plot which we subdivided into 400 1 × 1 m sub-plots at each site. At each sub-plot, and for each of our study periods within each year, we recorded floral production for each plant species present, as well as all visits observed of different pollinator species to the plant species present. To do this, during 3 1-h censuses per day, whenever we saw a pollinator enter our plot, we followed the sequence of visits it carried out, recording all the plant species it visited. We considered that a visit was successful if the pollinator species touched the reproductive parts of the plants. In addition, we recorded measures of fruit set for a subset of eight plant species (*Cistus salviifolius*, *C. crispus*, *C. ladanifer*, *C. libanotis*, *Halimium halimifolium*, *H. calcynum*, *H. comutatum*, and *Lavandula stoechas*), whose flowering period is mostly comprised within our study window (see for example Tobajas *et al.* 2023 for similar sampling efforts in the same study area). To do this, we marked open flowers within different individuals and fruits were collected once mature. For each individual (*N* = 542, 145 in 2020 due to issues with COVID.19 and 397 in 2021), we then calculated fruit set as the number of fruits produced within marked flowers/number of flowers marked.

There are several reasons why we focused on these species: (i) they are abundant species, where fruit set is relatively easy to measure, (ii) they are highly self-incompatible, and (iii) their flowers last for few hours, opening in the morning and losing petals in the afternoon (Bosch 1992), which allows to link the conditions of diversity, visitation frequencies received on one particular date to the reproductive success of those plants. All surveys were conducted within similarly sunny days with no wind. Although some of these shrubs can be included within two different 1 × 1m sub-plots, we considered their flowers and fruit production only within the sub-plot where the majority of the individual was located. Given that our focus is on functions related to the plant side of the interaction, plant reproductive success in this case, we considered each 20 × 20 m plot as a local community involving populations of different plant species, with the five different communities representing a region (which could be a meta-community in the case of particularly mobile species). For each community, we combined all the information from the 8 sampling periods per year and the sub-plots within plots (see Supplementary Appendix 2 Fig. S3 for examples of interactions between plants and pollinators at two sites).

We first focused on understanding temporal invariability across plant and pollinator communities, their interactions, and the resulting functions. Specifically, we assessed how temporal invariability for floral resource availability, pollinator visitation rates, plant–pollinator interaction frequencies and fruit set scaled across organizational levels: from populations, to communities and region (following Siqueira *et al.* 2023). To this end, we calculated temporal invariability as the inverse of the coefficient of variation (*1/*CV) across our two sampling years for each of these different system variables partitioned across three different lower-level components: populations, communities and the regional level. We understand that although two years of data is relatively small, particularly for inter-annual measures of stability, this exercise can still provide valuable insights. Particularly, this dataset, which continues to grow, can allow us to identify potential drivers of change, and use the data to generate hypotheses for future studies. Using the framework proposed by Wang *et al.* (2019a), we partitioned invariability in total regional (1/CV_*C,R*_) following the same nomenclature used by Wang *et al.* (2019a) floral availability, visitation rates, interaction frequencies and fruit set into two components, the temporal invariability of local communities (1/CV_*C,L*_) for each of these system variables and the spatial synchrony among these communities (Ψ_*C,L R*_). Local community invariability (1/CV_*C,L*_) was then further partitioned onto population invariability (1/CV_*S,L*_) and synchrony (Ψ_*S C,L*_) measures, such that CV_*C,R*_* = *CV_C,L_*Ψ_*C,L R*_ and CV_*C,L*_* = *CV_S,L_*Ψ_*S C,L.*_ In every case we calculated CV as the ratio of the variance of a given variable (flower availability, visitation rate, interaction frequency or fruit set) to its squared mean. Synchrony was calculated as the square root of the variance in flower availability, visitation rate, interaction frequency or fruit set divided by the squared sum of standard deviations in each case. All analyses were performed using the *var.partition* function and formulas from Wang *et al.* (2019a).

In addition, to assess the degree of variability in population sizes across different hierarchical levels within the region, we quantified the scaling relationship between variance and mean abundance. Specifically, to this end, we followed the methodology proposed by Taylor (1984) to quantify the relationship between the variance and mean abundance. The exponent *b* in Taylor’s power law equation, $Var=a*mean^{b}$ characterizes the scaling relationship between the variance and mean across different hierarchical levels within the region. To this end, we performed a log-log linear regression analysis on the mean-abundance and variance-abundance relationships, where the slope of the regression line represents the *b* value. A value of *b* = 0.5 shows a linear relationship between variance and mean, making the CV become a more intuitive measure of population variability, with values closer to zero indicating low variability relative to the mean and values closer to one indicating higher variability.

### Main results and discussion

**Figure 5.**
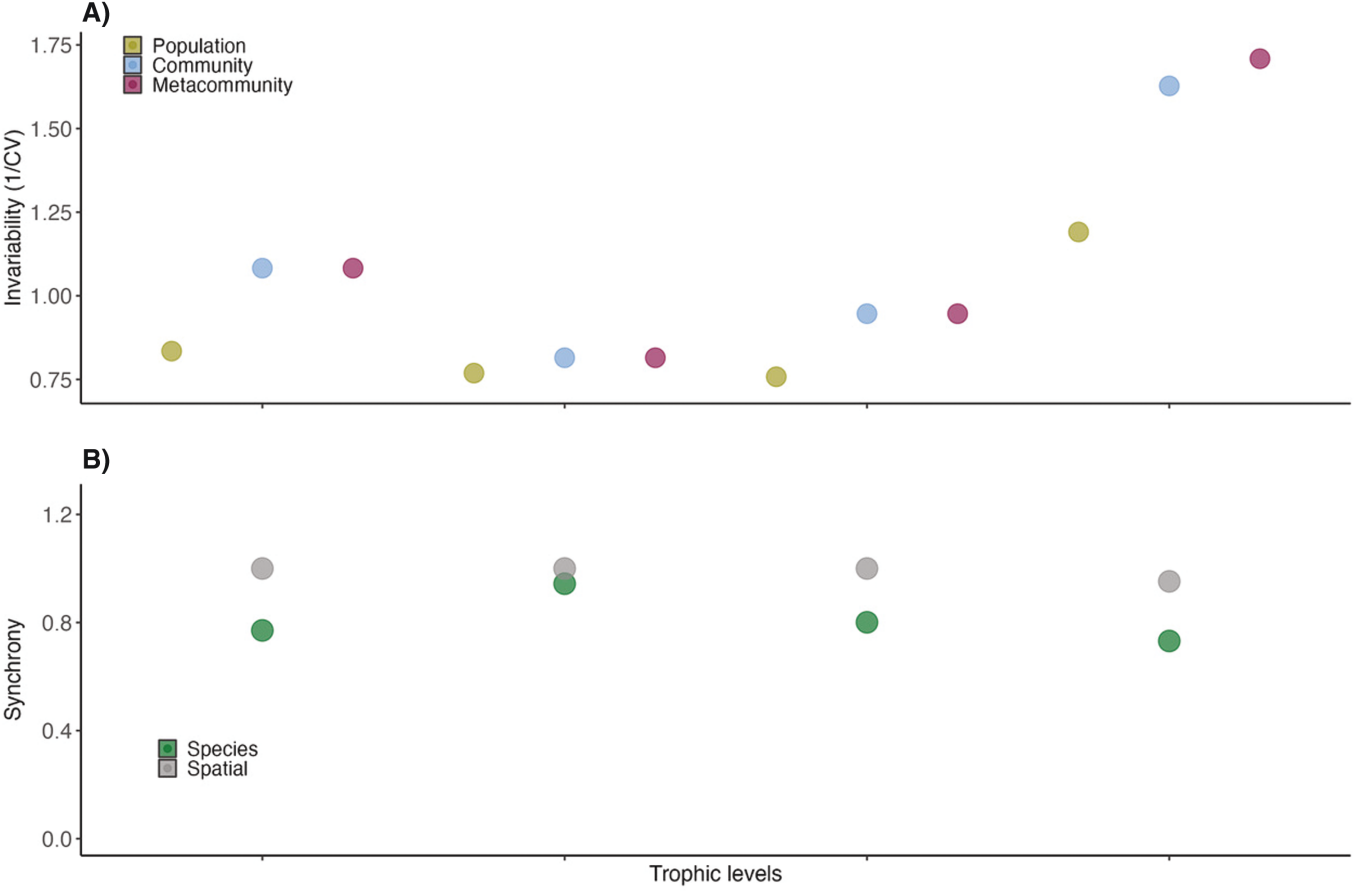
Scatterplots showing measures of (A) temporal invariability across organizational levels and (B) population and community synchrony values across the different system variables considered: flower availability, pollinator visitation rates, plant–pollinator interaction frequencies and fruit set.

Interestingly, temporal invariability in floral resource availability, pollinator visitation rates, plant–pollinator interaction frequencies and fruit set increased with increasing organizational level, in agreement with the patterns reported in previous experimental research for single trophic levels (Tilman 1995). This pattern is consistent across all of the properties considered, from floral production to pollinator visitation rates, plant–pollinator interactions frequencies and plant reproductive success (Fig. 5A), and is associated with lower species and spatial synchronies (Fig. 5B), and in general higher synchrony, population variability and community variability at the plant and pollinator trophic levels (Fig. 5). This pattern where stable communities are composed of more variable populations is a common pattern previously found in experimental (Lehman and Tilman 2000) and empirical settings (Siqueira *et al.* 2023).

Importantly, while floral resource production, pollinator visitation rates, and interaction frequencies exhibit high inter-annual variability, especially at the population level (within a given community), and previous studies have demonstrated significant turnover in community composition and structure over time within these communities (Magrach *et al.* 2023), fruit set displays notably less variation that is consistent across scales. Our results also show similar overall stability patterns across floral resource availability, pollinator visitation rates, plant–pollinator interaction frequencies and fruit set. This is in contrast with Siqueira *et al.* (2023), who analysed how stability scales across increasing trophic levels in freshwater food webs and found higher stability at higher trophic levels. Such disparity in the stability of mutualistic *versus* predator–prey interactions is difficult to disentangle given the limited nature of our current dataset (which we expect to grow in the future). However, potential mechanisms might include the way in which energy is transferred between trophic levels in food webs *versus* mutualistic networks. Food webs depict more hierarchical transfers of energy between trophic levels that involve several consumer levels where top consumers might display more stable populations compared to lower trophic levels because of their larger body size and high mobility, which allows them to alternate food resources, spatially couple energy channels and have access to the species and spatial asynchronous dynamics at the base of the local food webs (Rooney *et al.* 2006). Although these differences in mobility might also appear within pollination networks, these tend to be slightly more specialized than food webs (Thébault and Fontaine 2008).

This analysis is not meant to be an exhaustive exploration of the mechanisms determining the stability of plant–pollinator communities, as we are aware of the limitations of the relatively short time span of our data (eight sampling periods for two consecutive years), limited geographical cover (five sites), and focus on a single dimension of stability (intra-annual temporal invariability). Further, we acknowledge the limitations of our choice of metric, the CV, as a measure of invariability (Anderson *et al.* 2013; Segrestin *et al.* 2024). Specifically, the CV assumes that data follow a normal distribution, it can be sensitive to extreme values or outliers in the data, it can be influenced by the mean of the data, might not account for spatial or temporal autocorrelation patterns in the data and may not always adhere to Taylor´s power Law´s assumptions, particularly when the relationship between the variance and the mean is not linear or when other factors influence variability. In our data, our calculations of *b*, the exponent that quantifies the scaling relationship between the variance and mean show a close adherence to the theoretical expectation of *b* = 0.5 at the community and population levels (Supplementary Appendix 2 Table S1), although larger values at the regional level suggest a stronger-than-expected increase in variability with increasing mean abundance, potentially indicating non-linear scaling of population variance. Further, negative values of *b* imply that as the mean abundance increases, the variance tends to decrease, which is contrary to the typical expectation of Taylor’s law. This scenario might indicate some form of regulation or compensatory mechanisms within the population or community dynamics, where higher abundance levels lead to more stabilized variance, which warrants further investigation.

Nonetheless, our aim is to propose a way forward in our analysis of stability involving multi-trophic communities using currently existing theoretical frameworks used in other types of studies (Wang *et al.* 2019a), but explicitly including the interactions between two trophic levels (through visitation rates and realized interaction frequencies), which allows for a more thorough and complete analysis of the patterns of stability across spatial, organizational and trophic scales. As such, it serves us to illustrate the pattern observed in previous research focusing on single trophic levels, where population variability is relatively large but decreases with organizational levels due to compensatory and/or dominance responses among co-occurring species (Yachi and Loreau 1999, Fig. 4).

## Concluding Remarks and Future Research

Our review of the literature shows that our knowledge about the stability of plant–pollinator communities is fundamentally incomplete and limited mostly to single stability dimensions, individual spatial and organizational levels, numerical simulations and single trophic levels. To overcome these limitations, we have used an existing spatial stability framework and applied it to an empirical plant–pollinator dataset that integrates multiple spatial scales and organizational levels. Our analysis suggests that the stability (inter-annual temporal invariability) of plant–pollinator communities increases with the spatial scale, in agreement with classical single trophic level studies (Tilman 1995), and that this pattern is consistent across all organizational levels—from floral resources and pollinators, to species interactions and ecosystem functions.

A next step would be to investigate how plant, pollinator and interaction dynamics collectively contribute to overall temporal invariability in fruit sets. This involves adapting the framework developed by Wang *et al.* (2019a, Fig. 1A) to two trophic levels (Fig. 1B) and evaluate how invariability and synchrony across pollinator visitation rates, interaction frequencies and flower resource availability jointly contribute to the overall temporal invariability in fruit set (Fig. 1B). This can be done in two steps: (i) measure the contribution of local flower species diversity to pollinator visitation or interaction frequency invariability and synchrony (Fig. 1B), and (ii) quantify how the invariability and synchrony in interaction or visitation rates determine local fruit set invariability. In addition, the spatial turnover in floral resources should be explicitly considered as it can affect the dynamics of pollinator visitation or interaction frequencies across different communities, ultimately influencing fruit set stability at larger scales (e.g. through measures of beta-diversity, Fig. 1B). Existing datasets are still limited to study stability in ecological functions across organizational and trophic levels. Thus, future research on the stability of plant–pollinator communities would greatly benefit from including multi-annual data across different spatial (e.g. from sites to landscapes) and organizational levels (e.g. from populations to regions/metacommunities), various stability metrics (e.g. variability, resistance, persistence) and components of the community (e.g. from floral resources to reproductive success).

## Supporting Information

The following additional information is available in the online version of this article –

Appendix 1 includes articles used in the systematic map and Appendix 2 includes a flow diagram for a systematic map, a map of study sites and examples of plant–pollinator interaction networks.

plae026_suppl_Supplementary_File_S1

plae026_suppl_Supplementary_File_S2

## Data Availability

All data used within the paper and the code used to generate results can be found at https://github.com/amagrach/Stability.
