# Supplementary material for: Stability in plant–pollinator communities across organizational levels: present, gaps, and future
Source: AoB Plants. 2024 May 20;16(3):plae026. doi: 10.1093/aobpla/plae026 (PMC11151922; doi:10.1093/aobpla/plae026)
Supplement: plae026_suppl_Supplementary_File_S2 [file plae026_suppl_supplementary_file_s2.docx]

**Appendix 2.**

**Supplementary material.**

**
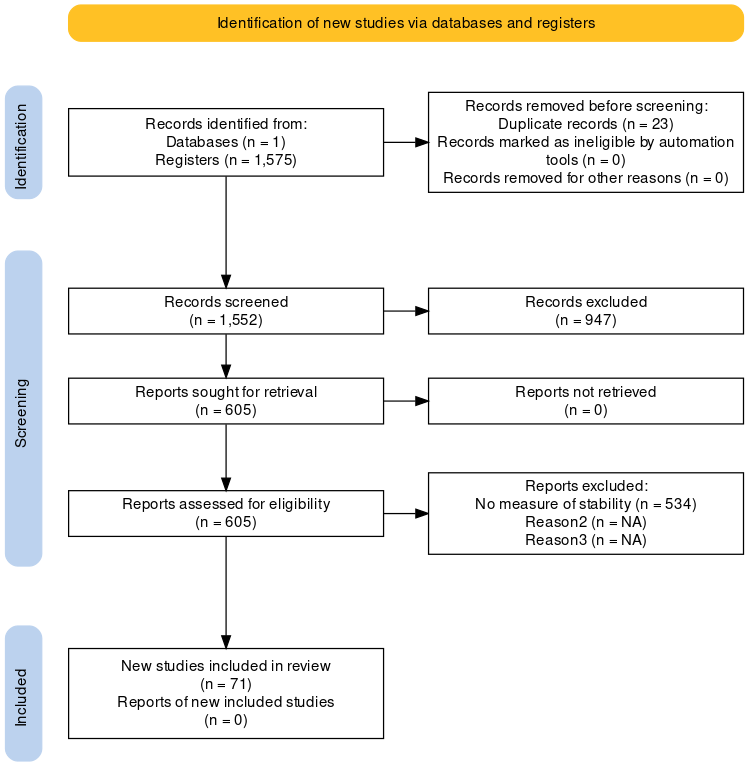
**

**Figure S1.** Flow diagram showing the steps followed to obtain the references for the systematic map.


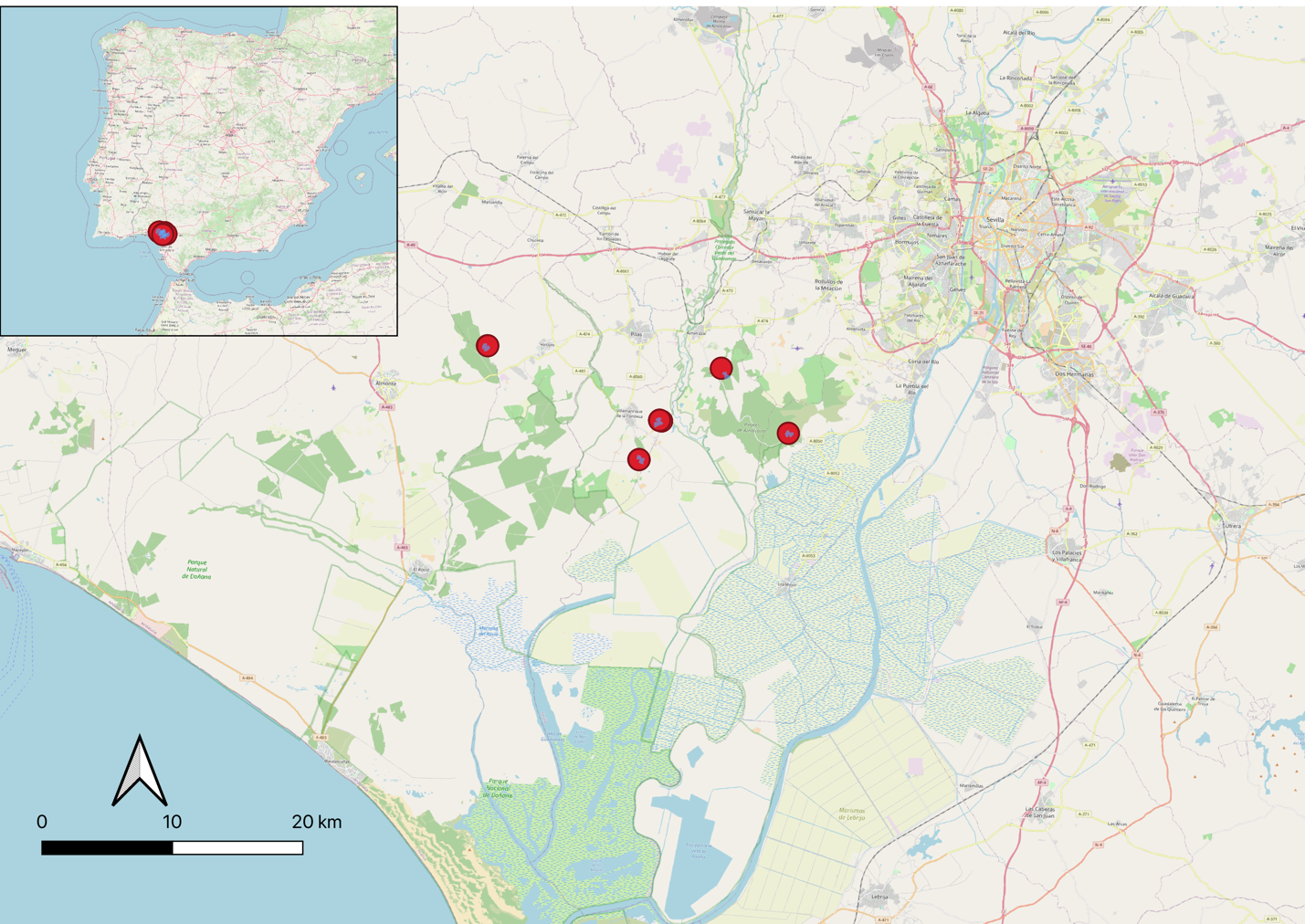


**Figure S2.** Location of the five study sites where information on floral resources, pollinator visitation rates, plant-pollinator interaction frequencies and plant reproductive success were collected multiple times throughout the flowering season for two consecutive years.


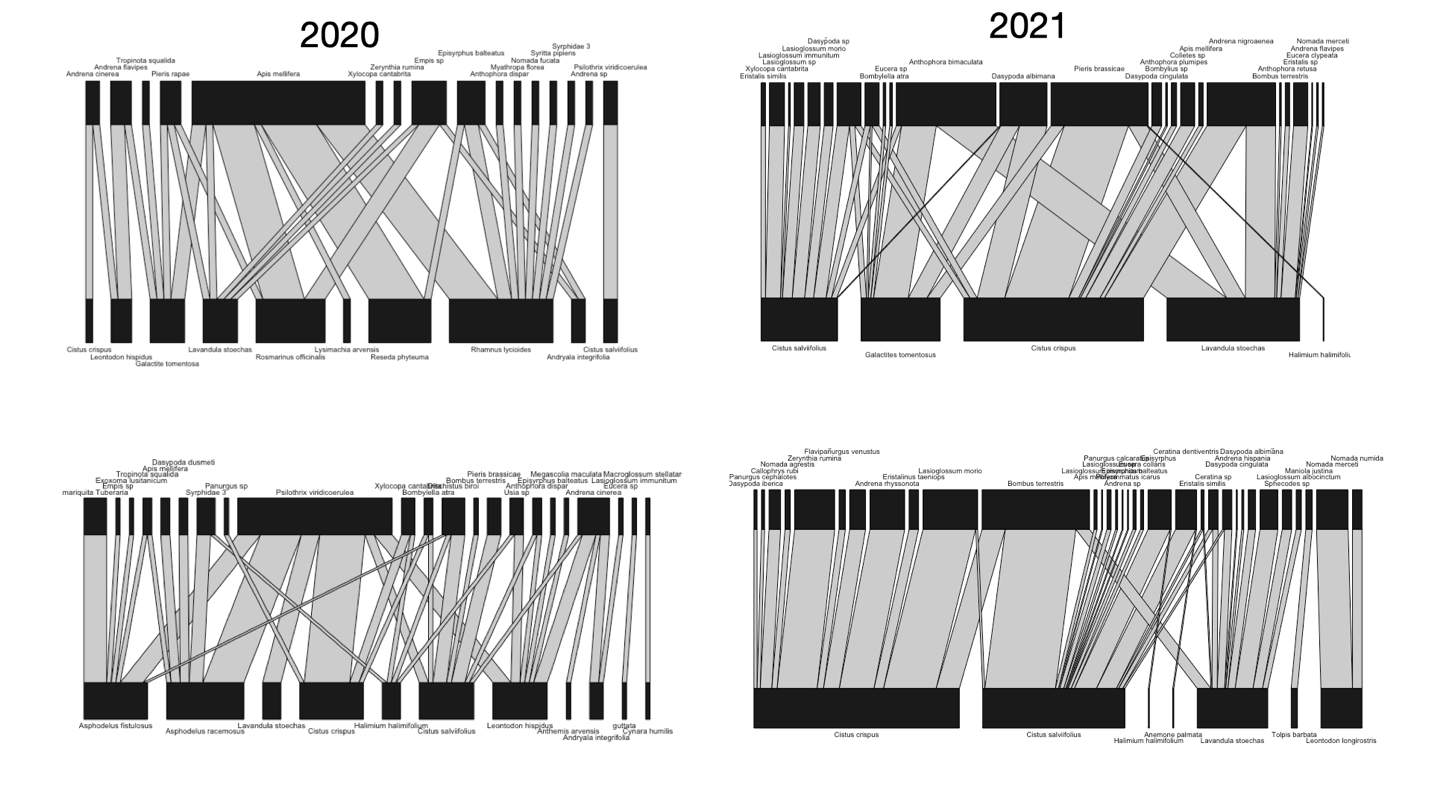


**Figure S3**. Bipartite graphs showing the frequency of interactions (size of lines) between plant and pollinator species at two of the sites surveyed for the two study years.


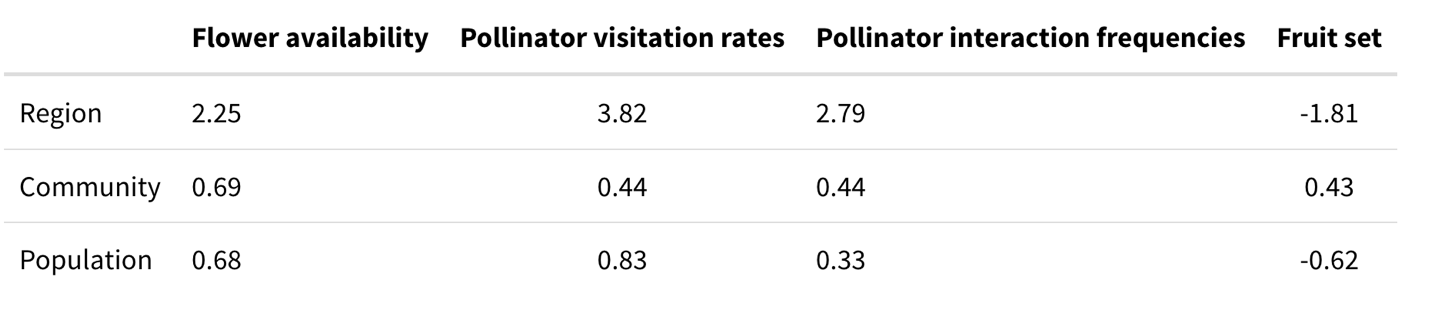


**Table S1**. Estimated b values for different hierarchical levels within the region. This table presents the estimated values of *b* – *b* representing the exponent in Taylor's power law – for various hierarchical levels within the metacommunity. The b values were calculated based on linear regression models fitted to log-transformed abundance data at each level. A *b* value close to 0.5 aligns with the expectation of Taylor's power law, indicating a quadratic relationship between the variance and mean abundance. Deviations from *b* = 0.5 may suggest alternative ecological processes influencing population variability. Negative values of *b* imply that as the mean abundance increases, the variance tends to decrease, which is contrary to the typical expectation of Taylor's law. This scenario might indicate some form of regulation or compensatory mechanisms within the population or community dynamics, where higher abundance levels lead to more stabilized variance, but would require further investigation.
